# KCO: Balancing class distribution in just-in-time software defect prediction using kernel crossover oversampling

**DOI:** 10.1371/journal.pone.0299585

**Published:** 2024-04-11

**Authors:** Ahmad Muhaimin Ismail, Siti Hafizah Ab Hamid, Asmiza Abdul Sani, Nur Nasuha Mohd Daud

**Affiliations:** 1 Department of Software Engineering, Faculty of Computer Science and Information Technology, University of Malaya, Kuala Lumpur, Malaysia; 2 Faculty of Computer Science and Mathematics, Universiti Malaysia Terengganu, Kuala Terengganu, Terengganu, Malaysia; Sunway University, MALAYSIA

## Abstract

The performance of the defect prediction model by using balanced and imbalanced datasets makes a big impact on the discovery of future defects. Current resampling techniques only address the imbalanced datasets without taking into consideration redundancy and noise inherent to the imbalanced datasets. To address the imbalance issue, we propose Kernel Crossover Oversampling (KCO), an oversampling technique based on kernel analysis and crossover interpolation. Specifically, the proposed technique aims to generate balanced datasets by increasing data diversity in order to reduce redundancy and noise. KCO first represents multidimensional features into two-dimensional features by employing Kernel Principal Component Analysis (KPCA). KCO then divides the plotted data distribution by deploying spectral clustering to select the best region for interpolation. Lastly, KCO generates the new defect data by interpolating different data templates within the selected data clusters. According to the prediction evaluation conducted, KCO consistently produced F-scores ranging from 21% to 63% across six datasets, on average. According to the experimental results presented in this study, KCO provides more effective prediction performance than other baseline techniques. The experimental results show that KCO within project and cross project predictions especially consistently achieve higher performance of F-score results.

## 1 Introduction

Software defect prediction (SDP) provides feedback on software defects that may only be detected in future software releases. To date, the extensive research on SDP has driven the involvement of more industries to participate in bringing more additional resources toward open-source software projects [1]. Hence, SDP research should thrive in the upcoming years with more public access software projects available. A software project in the context of SDP is a collection of procedures for the development of an intended software product with software versions by the related software artifacts. The software version contains an abundance of historic software project development information stored in code repositories. Just-in-Time SDP (JIT-SDP) enables the prediction process to be done once source code changes are committed to the code repositories. Specifically, JIT-SDP datasets require information regarding code changes through set of software metrics, defects information, and meta-information about the software project.

To model JIT-SDP, adequate training data should be available. Unfortunately, the required data is unavailable in the initial phase of software development. For this reason, the available datasets have a highly skewed distribution [2]. In this scenario, the clean class dominates the data set compared to the defect class. Lack of variation in SDP imbalance datasets and a lack of information on the distribution of the data are two of the key characteristics of SDP imbalance datasets [2, 3]. The imbalance in the class distribution of data leads to biases in the learning of the prediction model toward the clean class data. Consequently, the prediction model yields misclassification results. In such a scenario, the prediction model tends to be over-optimized for the clean class and may not generalize well to the defect class, leading to high misclassification rates. One way that researchers have tried to address the class imbalance problem involves oversampling techniques such as SMOTE [4], ROSE [5], ADASYN [6], and MWMOTE [7] to increase minority representation in datasets through the creation of synthetic samples based on existing samples. Under-sampling may also be used, which involves removing instances from the majority class in order to ensure that there are an equal or nearly equal number of samples. The disadvantages of undersampling include the risk of discarding valuable information and under-trained models [8]. In terms of addressing class imbalance in software defect prediction for information reservation, oversampling proved to be more effective than undersampling [9].

Oversampling presents a challenge since it introduces duplicate or overlapped instances into the distribution of the existing data [10]. Studies often rebalance samples by oversampling positive (defect) samples [11]. However, Zhang *et al*. [12] take the spatial distribution characteristics of samples into consideration, which cause the boundaries between different types of samples to become blurred. Several important aspects to consider when analyzing the spatial distribution of samples, including class imbalance severity, clustering, overlap class and distribution shape. For imbalance severity, a highly imbalanced dataset where the majority class significantly outnumbers the minority class produces class imbalance bias. As a result, minority class predictions are less accurate as the model tends to predict the majority class more frequently. Second, grouping or clustering instances belonging to the same class impacts the performance of a machine learning model. In densely grouped classes, the model has difficulty separating instances from those of other classes. In class overlap, the extent to which instances of different classes overlap or intermingle with each other. If instances are tightly clustered and overlap heavily, the model may have difficulty distinguishing between classes. Lastly, the distribution shape of the spatial distribution of instances across classes can also impact the performance of a machine learning model. For example, a dataset with instances spread evenly across a region may perform better than a dataset with instances tightly clustered in a few areas.

As motivation for the characteristics of spatial distribution, this study improves the ability to coop with these characteristics by proposing KCO. KCO offers an alternative solution to improve classification performance when dealing with imbalanced data. Integrating KCO with data pre-processing will enhance classification performance. Further, KCO avoids generating erroneous or duplicate data instances that lead to high false positives by avoiding generating less diverse data points within the minority class. Due to the above advantages, this study aims to create balanced class datasets while reducing redundancy and noise by increasing diversity of data In particular, we present KCO, which integrates the well-known KPCA as diversity analysis, spectral clustering to partition the spatial distribution, and crossover interpolation to generate new samples.

We conducted experiments on six large-scale software projects, namely Bugzilla, Columba, JDT, Mozilla, Platform, and PostgreSQL, which total 137,417 changes. We compare our technique with six baselines, including ADASYN [6], SMOTE [4], Borderline-SMOTE [13], MWMOTE [7] and MAHAKIL [11]. Several studies [14–16] suggest that random under-sampling (RUS) provides an effective sampling technique for JIT-SDP. This comparison also includes RUS. In the experiments, KCO achieved F1-scores of 42.2%, substantially higher than the baselines on average across the six projects.

This paper makes the following main contributions:

- We present the first consideration of KPCA in addressing the class imbalance issue for JIT-SDP, which has been neglected in previous studies.
- We develop a novel oversampling technique KCO for imbalanced datasets in JIT-SDP. Our proposed technique introduces the KPCA method to learn more representative feature representation of nonlinear distribution data and then the new instances of the defect data is generated by employing crossover-interpolation.

The remainder of this paper proceeds in the following manner. Section 2 presents a review of related work. Section 3 explains our technique framework. In Section 4, we describe the experimental setup. Section 5 presents the experimental results. Section 6 discusses threats to validity. Section 7 concludes the paper and presents the future work agenda.

## 2 Background

### 2.1 Just-in-Time software defect prediction

**Fig 1:**
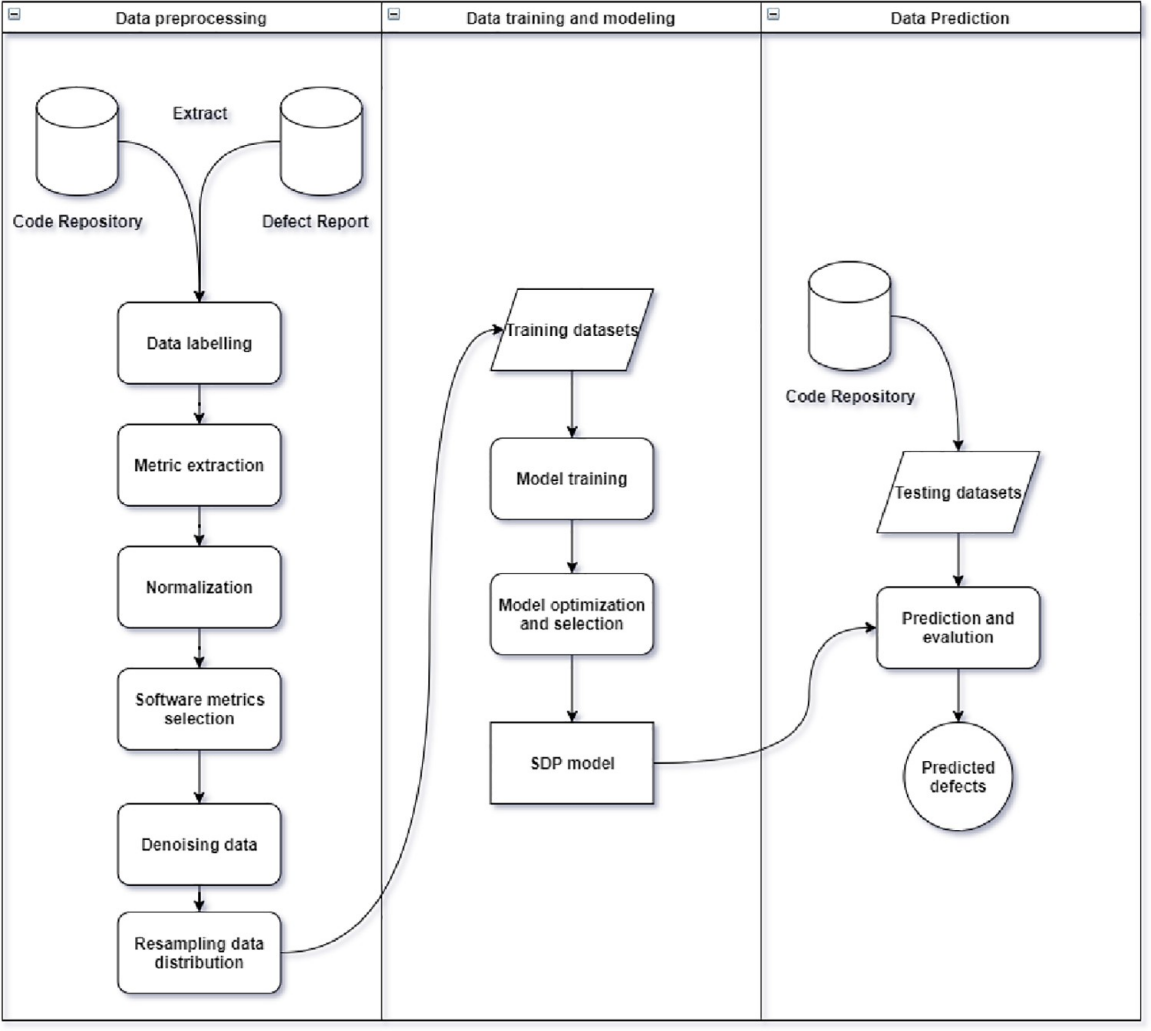
Workflow of JIT-SDP process.

SDP closely follows the project release schedule, based on code snapshots and defects found in previous releases. Therefore capable of predicting defect density and defect proneness at multiple prediction levels (modules, classes, changes) [17]. An alternative to prior prediction levels of SDP involves using software change histories to predict potential defects at the time of making changes to the repository (“just-in-time”), or JIT-SDP. The majority of software changes correspond to commits made to a Source Code Management (SCM) system or Version Control System (VCS). Fig 1 shows the overall JIT-SDP workflow. The workflow begins with identifying and assembling data sources that drive the model building process. The data sources include software code changes, issue reports, commit messages, and others from VCS and issue tracking system (ITS). The next step converts the raw data into feature vectors of software metrics. A feature vector obtained from software metrics by using metric filtering of collinearity analyses. Filtering analysis here is regarded as part of data pre-processing. In the following classification stage, model training and optimization take place in order to construct the prediction model. Following the construction of the prediction model, the model’s capabilities for predicting software defects will be evaluated. In recent years, many studies have focused on the application of deep learning techniques to JIT-SDP modelling [18, 19]. Yang et al. [20] proposed an approach called Deeper, which leveraged deep belief network and logistic regression classifier to predict defect-prone changes. Yang et al. [15] present various hybrid deep learning as ensemble models to see if it will improve the performance of JIT-SDP. In the study of Hoang et al. [21] utilize CNN to extract features from commit messages and code changes. Qiao and Wang [22] utilized neural network to select useful features for effort-aware JIT-SDP. Li *et al*. [23] leveraged an ensemble model of decision trees named EATT by using a greedy strategy to rank changes based on effort awareness. Similarly, Zhu et al. [24] introduced denoising autoencoder for features representation into CNN to construct the basic change features into the abstract deep semantic features. Motivated from these studies that conducted experiments on deep learning approaches is on the rise, in this work, we focus modelling of JIT-SDP using deep neural network algorithm.

### 2.2 Resampling approaches

Data from code changes often have class imbalance issues. Particularly, the difference between defect-inducing changesets and clean changesets has substantial implications for software projects. A typical distribution of software changes in a software project follows the Pareto Principle, which states that 80% of defects originate from by 20% of the code [24]. Accordingly, the ratio aligns with the observation that software defects in a project have skewed distribution, with a relatively small number of files or modules containing a significant number of defects. The class imbalance problem remains well-recognized as one of the major causes of the poor performance of defect prediction models [25, 26]. Random under-sampling (RUS) is the simplest and most common approach for undersampling in imbalance defect datasets [2, 14]. RUS approach randomly removes the majority class instances to match number of minority instances. Liu *et al*. [27] introduced an under-sampling approach based on sequential evaluation to guide the sampling process for subsequent classifiers. For each iteration, the majority of instances classified correctly by the current iteration will be excluded from consideration in the subsequent iteration. In the context of oversampling, Chawla et al. [28] proposed Synthetic minority over-sampling technique (SMOTE) as improvement technique of standard random oversampling. SMOTE randomly creates new samples between several instances within a defined neighborhood. He *et al*. [6] present ADASYN to assign weights to the minority classes and dynamically adjust the weights in a bid to reduce the bias in the imbalanced dataset. ADASYN incorporates a density distribution in automatically deciding the number of synthetic samples needed for each minority class sample. Barua *et al*. [7] proposed MWMOTE to divide positive samples into safety data, boundary data, and potential noise data, and then adopt different sampling strategies for different types of samples. MWMOTE adaptively assigns the weights to the selected samples according to their importance in learning. Cabral *et al*. [29] recently proposed oversampling rate boosting (ORB) to adjust the resampling rate over time rather than always using 1:1 ratio of the balanced defect dataset. ORB automatically readjusts the resampling rate that evolve throughout time according to the ratio of current instances class distribution. For the research community in class imbalance classification, SMOTE serves as a basis for oversampling. Numerous extensions and alternatives have been proposed since SMOTE was released in order to improve its performance in different situations [30]. Despite the fact that greater predictive impact for resampling on the minority class than on the majority class, most of the recent JIT-SDP works pre-processed the imbalance dataset by an under-sampling approach. The preference results from the fact that under-sampling requires shorter training time and a simpler process than oversampling [27]. Consequently, prior JIT-SDP research typically used under-sampling rather than oversampling [17]. For oversampling, JIT-SDP poses a challenge compared to other defect prediction especially in term concept drift which led to nonlinear data distribution [29, 31]. Addressing the challenges of oversampling in JIT-SDP requires the development of specialized techniques that account for the nonlinear data distribution, and complex interactions among software metrics.

## 3 Approach

In this section, we provide an overview of the proposed technique. Then, we describe diversity measurement to analyse data distribution. The next section discusses the partitioning of data for KCO. The final section contains details on crossover interpolation.

### 3.1 Overview of KCO

**Fig 2:**
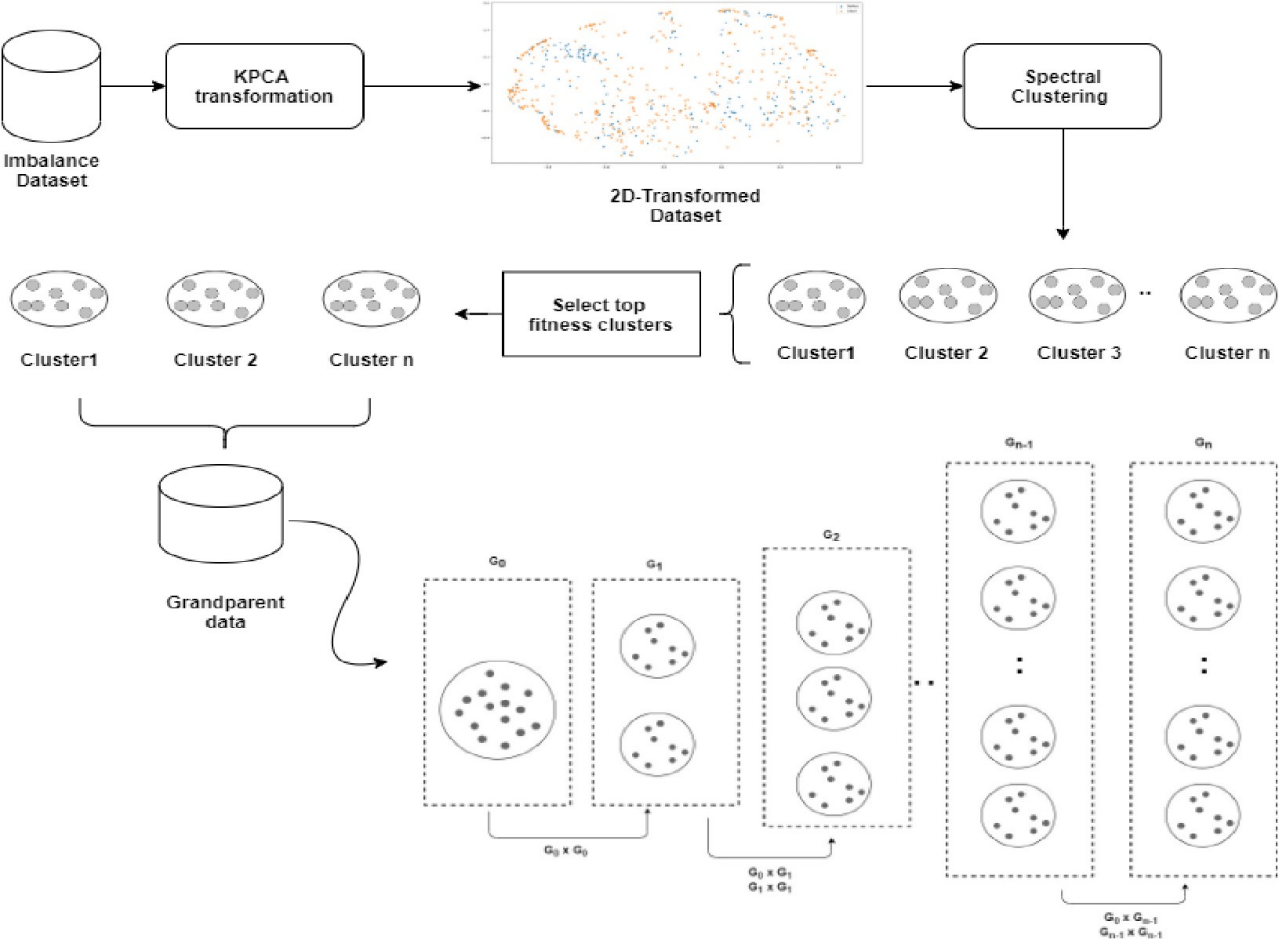
Workflow of KCO.

Using kernel analysis with spectral clustering and crossover interpolation as a combination method of oversampling, this study recommends Kernel Crossover Oversampling (KCO). The proposed algorithm consists of three phases designed to generate synthetic samples that possess both distinctive and common features, as illustrated in Fig 2. The first phase adopts KPCA to segregate the measurements for the minority samples. In this process, KPCA transforms the original dataset into a simpler dimension dataset to analyze the occupied space in the data distribution. In the second phase, spectral clustering divides the transformed data into several clusters. We then evaluate the fitness of each cluster based on the overlapped spatial distribution. By using the crossover operator of as in genetic algorithms, new samples are continuously synthesized to complete the oversampling of defect instances in the last phase. The newly generated samples combine with the initial data to produce a balanced dataset for training the JIT-SDP model. Algorithm 1 illustrates the full process. During Phase 1, steps 1 to 6 are described, then in Phase 2, steps 7 to 11, and finally, steps 12 to 22 for the last stage. To facilitate replication, we publish the source code of KCO at https://github.com/amuhaimin24/KCO—JIT-SDP. The following sections describe each phase in more detail.

**Algorithm 1** Pseudo Code of Kernel clustering oversampling (KCO)

**Input**: Dataset of majority and minority class samples *N*; desired balanced proportion *Pfp*

**Output**: Balanced dataset at a set *Pfp* value

**Procedure Begin**

 1) Split dataset *N* into majority class *N*_*maj*_ and minority class *N*_*min*_

 2) Compute the number of additional minority class to be generated *T* to attain *Pfp*

 3) *X*_*new*_: array for generated samples, initialized to 0

 4) *X*_*newchk*_: keeps count of the number of synthetic samples generated

 5) Compute Kernel function of PCA for dataset *N*, KPCA = KernelPCA(n_components = 2, kernel = ’rbf’) where n_components = dimension of data and RBF = radial basis function

 6) Transform dataset *N* into KPCA, *X*_*tranformed*_

 7) Create partitions of dataset *X*_*tranformed*_ using Spectral clustering technique, *cluster* = {*i*…10}

 8) For each *cluster*_*i*_, sequentially compute spatial distribution fitness *F*(*cluster*_*i*_) = *N*_*maj*_ / (*N*_*maj*_ *+ N*_*min*_)

 9) End for

 10) Rank clusters according to spatial distribution fitness in increasing order

 11) *Cluster*_*best*_: Select top three clusters

 12) While length of *X*_*newchk*_ ≤ size of *N*_*min*_

 13) Select samples *parent*_*a*_, *parent*_*b*_ from *Cluster*_*best*_, where *parent*_*a*_ and *parent*_*b*_ are not equal

 14) Generate a minority class synthetic sample *X*_*i*_ where *X*_*i*_ = average(*parent*_*a*_, *parent*_*b*_)

 15) Add *X*_*i*_ to *X*_*new*_ and increase *X*_*newchk*_ (i): *X*_*newchk*_ = *X*_*newchk*_ (i) + 1

 16)  End while

 17)  While length *X*_*newchk*_ ≤ *T*

 18) Select samples *parent*_*a*_, *parent*_*b*_ from *Cluster*_*best*_ and *X*_*new*_ respectively, where *parent*_*a*_ and *parent*_*b*_ are not equal

 19) Generate a minority class synthetic sample, where *X*_*i*_ = $\lambda ({parent}_{a})+(1-\lambda ){parent}_{b}$

 20) Add *X*_*i*_ to *X*_*new*_ and increase *X*_*newchk*_ (i): *X*_*newchk*_ = *X*_*newchk*_ (i) + 1

 21)  End while

 22)  Add *X*_*new*_ to dataset *N*

 23)  Return *N*

### 3.2 Diversity measurement

Euclidean distance fails to be effective in nonlinear distributions [32] as presented in JIT-SDP datasets. JIT-SDP data typically exhibit a nonlinear distribution as a result of the uncorrelated relationship between software metrics. Several factors may affect the distribution, including clusters, non-convex shapes, or overlapping regions that are not accurately represented using a linear distance measure. The relationship between data points is unable to be well-represented by a straight line calculated by Euclidian distance. Therefore, the measure does not accurately reflect the diversity of data points. Moreover, the JIT-SDP datasets contain noise or duplicates as a result of the collection process for the metrics [2]. Accordingly, the Euclidean distance measure unable to identify highly correlated or duplicated data samples within nonlinear distribution which failed to provide meaningful during information classifier training. As an alternative to handle highly correlated data, one may utilize feature engineering techniques such as Principal Component Analysis (PCA) [33]. PCA learns the original feature combinations linearly in new dimensional spaces. Nevertheless, PCA assumes that the learning data follow a linear separable Gaussian distribution. For real world data, particularly code changesets, linearly separated data is impractical due to the nonlinear structures of software metrics.

Prior studies have indicated that KPCA perform better than PCA for software engineering tasks [34]. Researchers have investigated the use of KPCA in software defect prediction, especially for the selection of features. Xu *et al*. [35] found that basic classifiers including KCPA as a feature selection method achieve promising performance when compared to 41 baseline methods. Experimental results indicate that the framework outperforms PROMISE and NASA datasets, particularly in terms of F-measure, MCC, and AUC. Ho *et al*. [36] utilized KPCA to reduce the dimensions of defect feature spaces from software metrics in order to extract essential information. A deep neural network (DNN) is then built to emphasize the semantic relations between software metrics so that defect data are distinguished from non-defect data using newly generated features from KPCA. Azzeh *et al*. [37] examine the performance of nonlinear kernel functions and linear kernel functions in the context of different experimental parameters such as the granularity of the data, the imbalance ratio of the dataset, and feature subsets. According to their findings, RBF is the only kernel function that exceeds linear and other nonlinear kernel functions. Nonetheless, reducing the dimensionality of a dataset did not often improve the accuracy of software defects prediction [38, 39]. Therefore, the KPCA should not be limited to measuring the similarity between features in software metrics. In other aspects of JIT-SDP, KCPA presents a promising alternative. As a result of KCPA, patterns in the data are identified that are not apparent by traditional methods of data representation, including handling high-dimensional datasets and capturing non-linear relationships among features. Therefore, the analysis of data distribution can be particularly important for data resampling.

This study employs KPCA to map multivariate of software metrics into a linear projection using a nonlinear kernel function. The process of data projection involves transforming the original data into lower dimension data. Data transformation process converts multivariate data into a new set of uncorrelated variables. Enabling efficient multidimensional scaling of JIT-SDP datasets with varying software metrics. In this way, the diversity analysis of JIT-SDP datasets by KPCA is independent of the data dimensions and becomes a scale-independent measurement. Therefore, the complex structure becomes easier to manage and allows the representation of features to be projected in a linear manner. Using a Radial Basis Function (RBF) kernel, KPCA provides a linear representation of the data while preserving the relative distances between pairs of data points that are close to the original space.

Algorithm 2 Pseudo Code of KPCA

Input: Dataset *N* x *D*; Number of principal components: *k*

Output: Projected_data with an *N* x *k* matrix representing the projected data onto the selected principal components

Procedure Begin

 1) Compute Kernel Matrix *N* x *N*, K

 2) For i = 1 to N do

 3) For j = 1 to N do

 4)  K[i, j] = RBF(Data[i], Data[j])

 5) Center the Kernel Matrix

 6) for i = 1 to N do

 7)  for j = 1 to N do

 8)   K[i, j] = K[i, j] - mean_rows[i] - mean_cols[j] + mean_total

 9) Compute the Eigenvectors and Eigenvalues of the centered kernel matrix K

 10) Sort the eigenvalues in descending order and select the top k eigenvalues and corresponding eigenvectors

 11) Project Data onto Principal Components

 12) for i = 1 to N do

 13) for j = 1 to k do

 14)  Projected_Data[i, j] = dot_product(alpha[j], K[i, :])

 15) Return *Projected_Data*

### 3.3 Data partitioning

**Fig 3:**
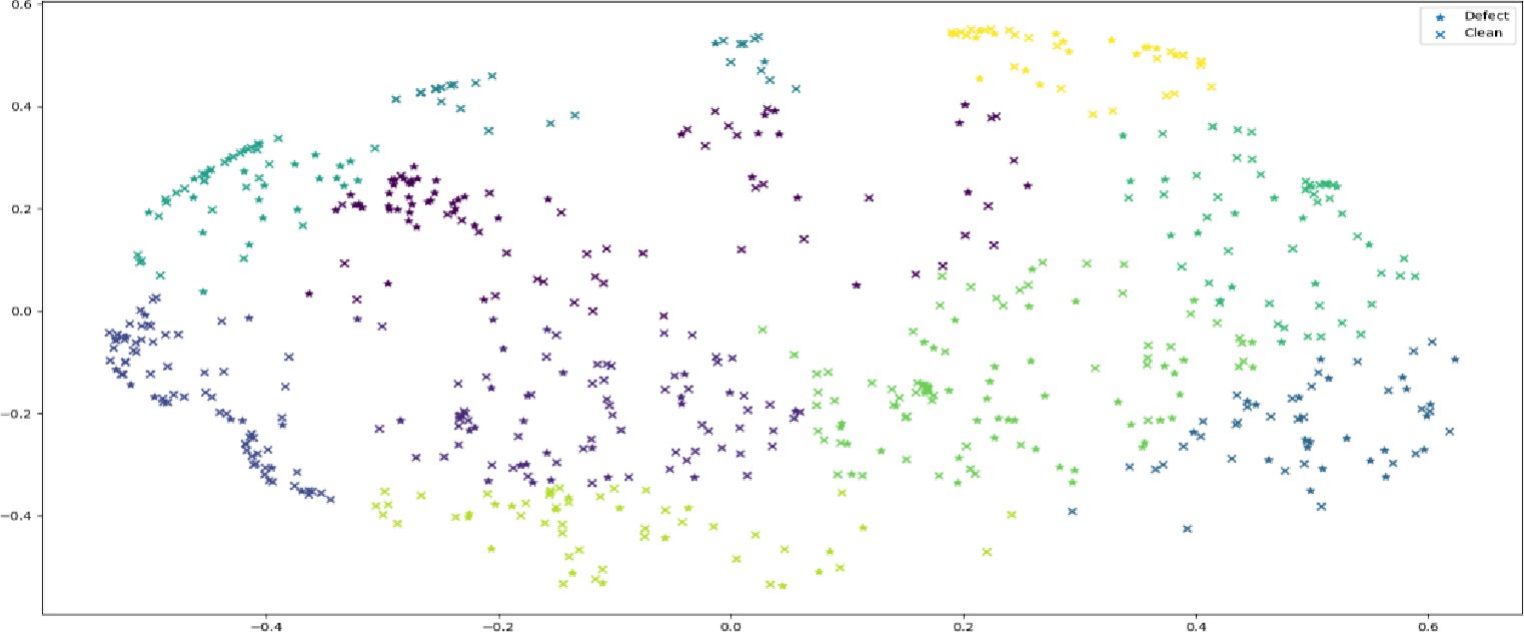
Spectral clustering within transformed data.

KCO relies on spectral clustering to simplify multidimensional nonlinear datasets while reducing them into clusters of data exhibiting similar characteristics on lower dimensions. Spectral clustering treats the data clustering problem as a graph partitioning problem without making any assumptions about the shape of the clusters. Fig 3 illustrates spectral clustering in sample distributions in the JIT-SDP dataset.

The basic premise of spectral clustering in defect datasets is as follows: For a dataset with *n* samples *D* = {x_1_, x_2_, …, x_n_} and each sample consist of variables x_i_ = {v_1_,v_2_,..,v_m_}, where *m* is the number of software metrics. The clustering is based on dividing each sample into *k* clusters C = {C_1_, C_2_,.., C_k_}. As a result, the samples in the clusters have a variance that is:

$${argmin}_{s}\sum_{i=1}^{k}\sum_{x\in C_{i}}\|x-\mu_{i}\|\#(1)$$ (1)

Where, *μ_i_* is the mean value of the samples in *C_i_*.

Fitness calculation is made for each cluster based on the number of samples taken from both the minority and the majority classes. The intuition behind fitness assessment for clusters is that regions with a lower proportion of majority samples should have fewer overlapped spatial distributions. The following formula calculates the fitness weight of each cluster:

$$Fitness(C_{i})=\frac{Length(X_{maj})}{Length(X_{maj}+X_{min})}$$ (2)

A fitness evaluation is conducted for each cluster, and the three best clusters are chosen. Clusters selected for interpolation have a greater proportion of empty spaces, indicating that there are more areas that can be utilized for new samples. Using selected clusters, a pool of suitable templates for oversampling is established.

### 3.4 Crossover-interpolation

Interpolation in oversampling generates synthetic samples from existing minority class samples. One of the earliest methods for oversampling was the SMOTE, introduced by Chawla *et al*. [4]. SMOTE uses interpolation to generate synthetic samples from existing minority class samples. Even so, the use of SMOTE to develop prediction models may still result in overgeneralization as it relies solely on the selection of nearest neighbor instances. Due to the limitations of SMOTE, a variety of modifications have been proposed, including Borderline-SMOTE [13] and MWMOTE [7]. Nevertheless, prior techniques unable to provide a diverse and balanced set of synthetic samples from datasets with high-dimensional input features. Cross-over interpolation provides an alternative way to generate synthetic samples by combining or "crossing over" the features of two existing minority class samples. Consequently, the generation new samples exhibit more representative and diverse to better reflect minority class distributions. In SDP, Bennin *et al*. [11] first to propose crossover interpolation into oversampling process which named as MAHAKIL. They adopted Mahalanobis distance to rank instances and divide them into two groups. During the generation of new instances, two corresponding instances are chosen from each group. Synthetic instances tend to be more diverse when pairs of selected instances do not have a close distance between them. In comparison to SMOTE-based oversampling techniques, MAHAKIL offers superior performance and greater stability. Even though MAHAKIL intends to improve the diversity of data, it unable to detect defects consistently, thereby reducing its value. Particularly, MAHAKIL ineffective to calculate the Mahalanobis distance when the number of minority class instances is smaller than the dimensionality of these instances. Thus, MAHAKIL unable to function optimally when the number of minority class instances is less than the number of metrics. Zhang *et al*. [12] extended the work of Bennin *et al*. [11] by adding K-means clustering to MAHAKIL in order to improve the recognition rate of positive samples. By using K-means, they divide positive samples into K-clusters and crossover interpolate synthetic instances.

This study uses the crossover operator to generate new samples in the same manner as genetic algorithm. In this process, chromosome information contributes by two parents to generate a child. Chromosome information defined in this study as software metrics for JIT-SDP modeling purposes. In order to generate new samples, crossover operators combine the characteristics of two samples. Given two samples of $S_{a}^{g}$ = [*a*_1_,…, *a*_*l*_] and $S_{b}^{g}$ = [*b*_1_,…, *b*_*l*_] are two chromosomes crossed in *gth* generation and *l* is the length of chromosome or features, the child sample of *g* + 1 *th* generation is:

$$S_{c}^{g+1}=\lambda S_{a}^{g}+(1-\lambda )S_{b}^{g}$$ (3)

Where *λ* provides a random variable between a range of [0,1].

**Fig 4:**
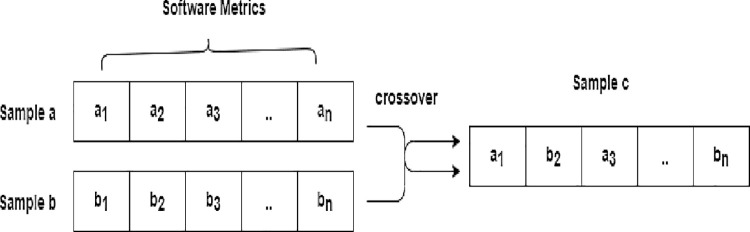
Example of multi-point crossover.

During the experiment, λ is set to 0.5 for generating the child samples. The setting means that the child samples inherit 50 percent of their characteristics from each of their parent samples. Fig 4 illustrates an example of crossover operation during the generation of a new sample.

**Fig 5:**
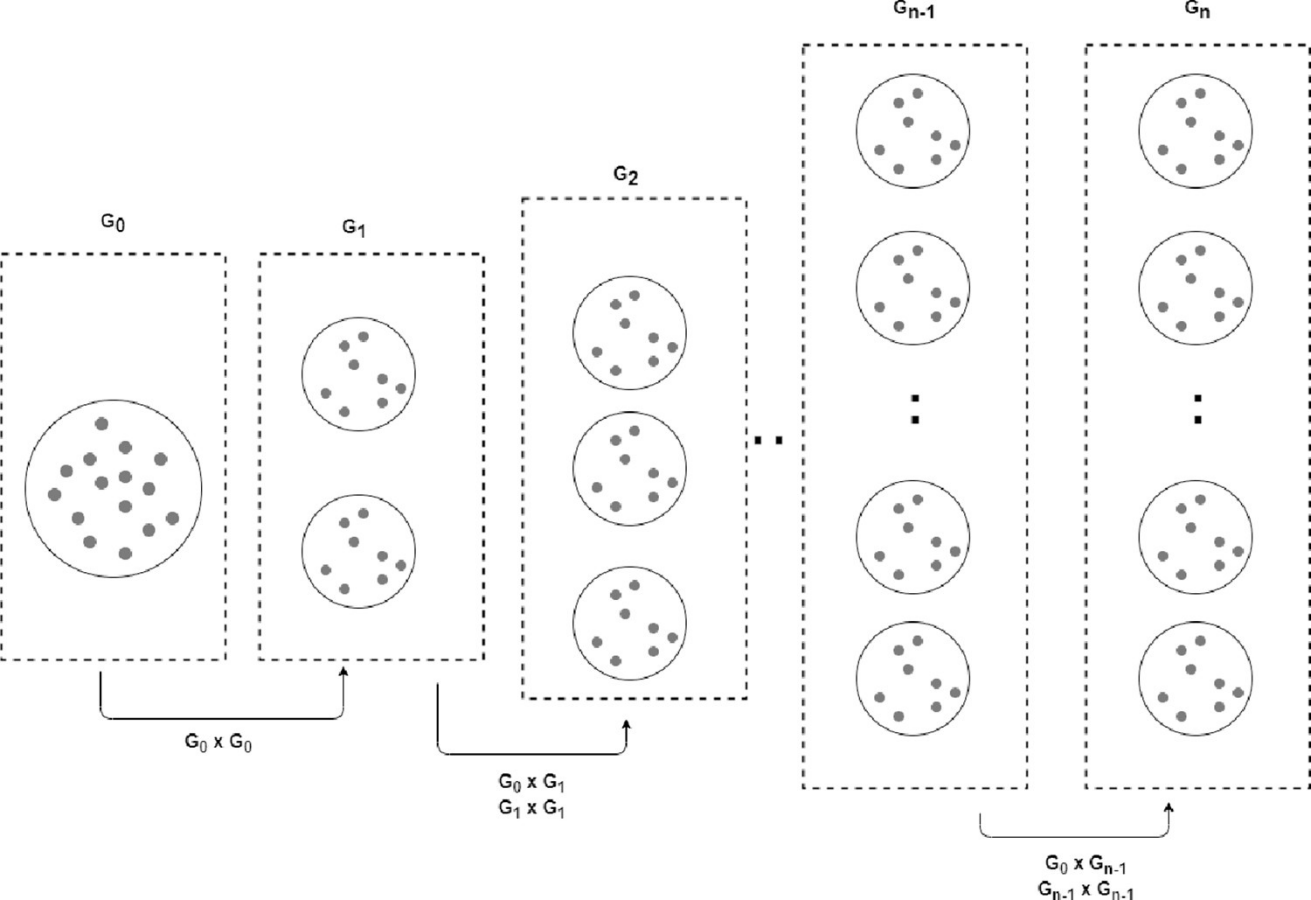
Crossover interpolation across generations.

Fig 5 illustrates the generation of new synthetic samples based on the level of inheritance. First, based on diversity measurements obtained from KPCA, the grandparent samples are identified, *G*_0_. The samples from *G*_0_ are then used to generate the *G*_1_ set of new synthetic samples. To prevent new samples from entering the region of the majority class, the first parent node or grandparent act as a boundary such that all children produced in the future reside within the range of the parents. In the second generation *G*_2_, samples from grandparent and samples from *G*_1_ are selected as template to generate new samples. In case of the interpolation at current generation is still not meet with the maximum samples, the process continues to crossover interpolate the samples within the previous generation until maximum number reach. The process of pairing the child nodes with older generations is repeated until the generated samples are sufficient (greater than or equal to the required number of samples). The pairing process is carried out using the sequential information inherited from the immediate parents of the instances beginning at *G*_1_.

## 4 Experimental setup

In this section, we evaluate the effectiveness of KCO. The experimental environment is an Intel(R) Core (TM) I5-10400 2.9 GHz CPU, 16 GB RAM desktop running Windows 10. In Sections 4.1 and 4.2, we describe the experiment setup and evaluation metrics, followed by the presentation of three research questions in Section 4.3, along with the experimental findings that answer these research questions. Artificial neural network algorithm serves as the classifier algorithm of the JIT-SDP model in this comparison. The classifier for defect prediction utilizes the resampled data generated by resampling techniques. For comparison, we use default hyperparameters for all compared techniques.

### 4.1 Benchmark datasets

We evaluate six imbalanced software project datasets which comprise Bugzilla, Columba, Eclipse.JDT (JDT), Eclipse.Platform (Platform), Mozilla, and PostgreSQL (Postgres). Note that all the datasets are imbalanced. The most imbalanced dataset, Mozilla, contains only 5% defects, while the most balanced dataset, Bugzilla, contains 36% defects. To ease the analysis of prediction results, these datasets are classified into two severity groups as shown in Table 1. Mild imbalanced class refers to datasets that contain more than 25% defects. High imbalanced datasets are based on datasets with fewer than 25% defects. The severity of the imbalance class represents the difficulty for data resampling in the imbalance distribution.

**Table 1:** Imbalanced class datasets.

Project	Project Duration	# Instances	Defect %	Severity
Columba	08/1998–12/2006	4455	31	Mild imbalanced class
Bugzilla	11/2002–07/2006	4620	36	Mild imbalanced class
Postgres	11/2002–07/2006	20431	25	Mild imbalanced class
JDT	05/2001–12/2007	35386	14	High imbalanced class
Platform	07/1996–05/2010	64250	14	High imbalanced class
Mozilla	08/1998–12/2006	98275	5	High imbalanced class

### 4.2 Evaluation measures

The evaluation measure is important to reveal the performance of the classifier, especially for imbalanced datasets. Some conventional measures lead to a wrong conclusion owing to the skewness of the class distribution [10]. For example, consider an extremely imbalanced dataset: 99% of instances are of the majority class, and the remaining 1% samples belong to the minority class. In case of using the accuracy measure which indicates how many test samples are correctly classified as the evaluation criterion, even if the classifier ignores all of the minority classes, it still reaches a very high accuracy rate of 99%. Therefore, this experiment also considered F1-score, which is a commonly used measure to evaluate classification performance. F1-score combines Precision and Recall from a confusion matrix. The confusion matrix lists all four possible prediction results. If an instance is correctly classified as a defect, it is a true positive (TP); if an instance is misclassified as a defect, it is a false positive (FP). Similarly, for false negatives (FN) and true negatives (TN). Based on the four numbers, Precision, Recall, and F1-score are calculated. Precision is the ratio of correctly predicted defect instances to all instances predicted as defects (Precision=TP/(TP+FP). Recall is the ratio of the number of correctly predicted defect instances to the actual number of defect instances (Recall=TP/(TP+FN). Finally, F1-score is a harmonic mean of Precision and Recall, $Fscore=\frac{1.25\times Recall\times Precision}{\left(0.25\times Precision+Recall\right)}$.

### 4.3 Experimental design

The main goal of the research is to propose a resampling technique named KCO on JIT-SDP datasets. To that end, we conducted experimental investigations based on three research questions in the following order.

***RQ1*: *Does KCO contribute to the diversity of the datasets*?**

To evaluate whether KCO improves the diversity of the data distribution, we conduct an analysis to compare the distribution of the resampled data by oversampling when applied separately to oversample datasets. The analysis considers sparsity of data distribution where the larger sparsity data contain less information across data classes. Oversampling in training datasets reduces the impact of noise and improves sparsity of data distribution toward dense data. Dense data yields more informative data, which results in more accurate predictions due to more data available for model training. By measuring the percentage of non-zero values in the data, sparsity of the data distribution is analyzed using data density measurement. The analysis provides a simple and straightforward way to quantify the sparsity of the data. In measuring the diversity of data for resampled datasets (*d*), sparsity formulation is utilized. $sparsity=1-\frac{non\_zero\;(d)}{size(d)}$.

***RQ2*: *Does KCO improve the diversity within the data distribution at the expense of its ability to provide accurate predictions*?**

To validate the effectiveness of oversampling providing diverse data distribution, we compare the proposed technique with baseline techniques. Evaluation of accuracy prediction is conducted based on 10-folds stratified within project validation. The validation started with the splitting of data into 8:2 ratio, for both training and prediction datasets. Then, the training dataset undergoes 10-fold stratified within project validation. The datasets are divided randomly into 8-folds, 2-folds serve as training data, and the remaining fold serves as test data. In stratified cross validation, each fold is used as a testing dataset only once. Additionally, the data are folded so that every fold consists of the same proportions as the original dataset. the data need to be folded in such a way that each fold consists of the same proportions as the original dataset. The average result is recorded using StratifiedKFold to strengthen the reliability of the experiment outcomes.

***RQ3*: *How does KCO addresses class imbalance problem under different imbalance severity*?**

To evaluate the performance of oversampling more accurately, the effectiveness of oversampling should be evaluated in the context of the different validation settings. We conducted cross-project prediction and timewise prediction. In cross project prediction, the prediction of software defects is evaluated across different software projects. Specifically, the models are constructed by one source of software project and use these models to predict software defects on another target software project. For a set of *n* projects, this method produces *n* * (*n*—1) prediction effectiveness values. In timewise prediction, we evaluated JIT-SDP within the same project datasets, in which the chronological order of changes based on the commit date is considered. Assuming the changes are divided into *n* parts, we first construct the models based on the changes from part *i* and *i* + 1. Then we use the constructed models to predict the changes from part *i* + 4 and *i* + 5. For each fold, it consists of varies in the size of data and imbalance ratio.

## 5 Experimental results

RQ1: Does KCO contribute to the diversity of the datasets?

Table 2 provides data distribution for the different resampling techniques. The results indicate KCO achieves the lowest sparsity values across all datasets. Considering the difference in sparsity values, only KCO provides a significant difference value of 8% to 10% for data sparsity before resampling (original). In the case of Mozilla, the data sparsity generated by KCO, MWMOTE, and MAHAKIL is similar.

**Table 2:** Distribution of software project data after data resampling.

Techniques /Datasets	Columba	Bugzilla	Postgres	JDT	Platform	Mozilla
KCO	23%	18%	20%	22%	22%	18%
MAHAKIL	28%	20%	22%	24%	25%	18%
MWMOTE	30%	24%	22%	26%	26%	18%
Borderline	32%	26%	26%	29%	29%	21%
RUS	32%	25%	25%	31%	29%	20%
SMOTE	32%	25%	25%	30%	29%	22%
ADASYN	33%	26%	26%	31%	30%	23%
Original	34%	26%	28%	33%	34%	28%

Resampling low sparsity datasets becomes more difficult due to less significant variation among data points within the dataset, making them dense datasets. Among the datasets, Bugzilla exhibits the most dense distribution. As a result, baseline resampling techniques, including SMOTE, Borderline, RUS, ADASYN and MWMOTE fail to significantly improve data sparsity. Indeed, resampling in a dense dataset presents difficulties in generating more data samples in limited empty spaces. On the contrary, KCO provides better data distribution than baseline techniques with more robust performance in identifying empty spaces by using kernel function. Overall, KCO produces more sparse data than SMOTE, Borderline, RUS, and ADASYN. KCO compares favourably with data generated by MAHAKIL and MWMOTE utilizing Mozilla, Bugzilla, and JDT. Considering that KCO generates more diverse data than other baseline techniques, contributing to data distribution diversity.

Having more diverse data distribution is preferable because it can help improve the performance of machine learning models. Diversity in the training data ensures that the training data can provide more discriminative information for the model. Such diverse distribution provided by KCO is preferable because it improves the generalization capabilities of the classifier, enhances its ability to handle complex class boundaries, increases discriminative power, and addresses biases toward certain intra distribution. By incorporating diverse data distribution for model training, defect classifier become more robust, adaptable, and capable of making accurate predictions on a broader range of data instances.

RQ2: Does KCO improve the diversity within the data distribution at the expense of its ability to provide accurate predictions?

**Fig 6:**
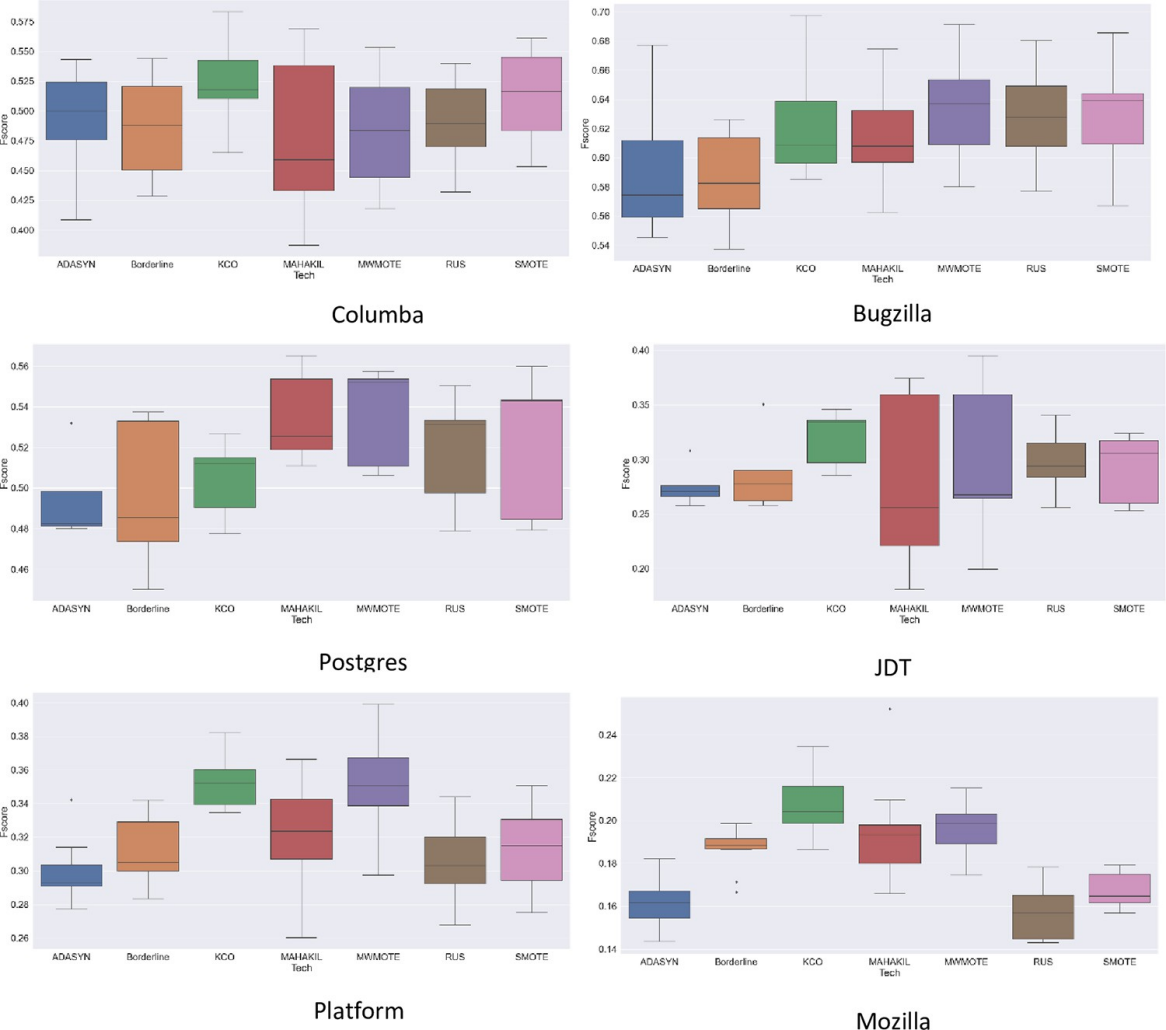
Prediction performance in different training/testing folds of within project prediction.

Based on Table 3 and Fig 6, KCO, MAHAKIL, and MWMOTE in general are the top performance techniques which outperform other baseline techniques in terms of F-score measure for almost all datasets. Surprisingly KCO achieved the highest performance among them, especially in the severely imbalanced datasets as in Platform and Mozilla. On average, KCO achieves 52.6%, 32%, 35.2%, and 20.7% of the highest average F-score in Columba, JDT, Platform, and Mozilla respectively. RUS is recognized as the most commonly used resampling technique for imbalanced datasets, however its F-score consistency is almost equal to other oversampling techniques such as ADASYN, SMOTE, and Borderline.

**Table 3:** Prediction performance of F-score for within project prediction.

Techniques /Datasets	ADASYN	BORDERLINE	MAHAKIL	MWMOTE	RUS	SMOTE	KCO
Columba	49.2	48.6	47.9	48.5	49.1	51.2	**52.6**
Bugzilla	58.9	58.6	61.1	**63.4**	62.9	62.7	62.5
Postgres	49.5	49.6	53.5	**54.6**	51.8	52.2	50.4
JDT	27.6	28.8	27.8	29.7	29.8	29.2	**32.0**
Platform	30.0	31.2	32.0	34.1	30.6	31.2	**35.2**
Mozilla	16.1	18.6	19.4	19.5	15.7	16.8	**20.7**
Average	38.6	39.2	40.3	41.6	40.0	40.6	**42.2**

For stability, consideration should be given to the robustness of the techniques in dealing with severely imbalanced datasets. The severity of the imbalanced ratio certainly affects the stability of resampling techniques. Due to the severity factor, the resampling techniques have difficulty to achieve consistency of F-scores when dealing with large datasets as presented in Platform and Mozilla. Oversampling techniques with data partition embedded algorithms such as MAHAKIL, MWMOTE, and KCO tend to achieve a better F-score for these datasets, especially KCO which achieves the highest average score for all severely imbalanced datasets. The results show that simple techniques such as RUS, SMOTE, and ADASYN fail to maintain stability when dealing with imbalanced datasets in JIT-SDP. Contrary to KCO, the results show the most effective resampling technique when dealing with highly imbalanced data. KCO provides a more diverse distribution of data especially for severely imbalance datasets. Specifically, the generation of new synthetic data through multiple levels of inheritance from the original data, improving the diversity of the overall data distribution. The prediction model can learn more discriminative patterns and make better-informed decisions, resulting in improved prediction performance. In contrast to mild imbalanced datasets, KCO fails to provide a reliable result because defect class have dense distributions. Consequently, KCO faces a challenge in conducting diversity analysis through KPCA. For other baseline techniques, mild imbalance datasets prove easier to resample the class distribution considered dense. The main factor is that through diversity measures (Euclidian distance and Mahalobis distance) by baseline techniques can provide meaningful attributes that effectively distinguish classes.

*RQ3*: *How does KCO addresses class imbalance problem under different imbalance severity*?

**Fig 7:**
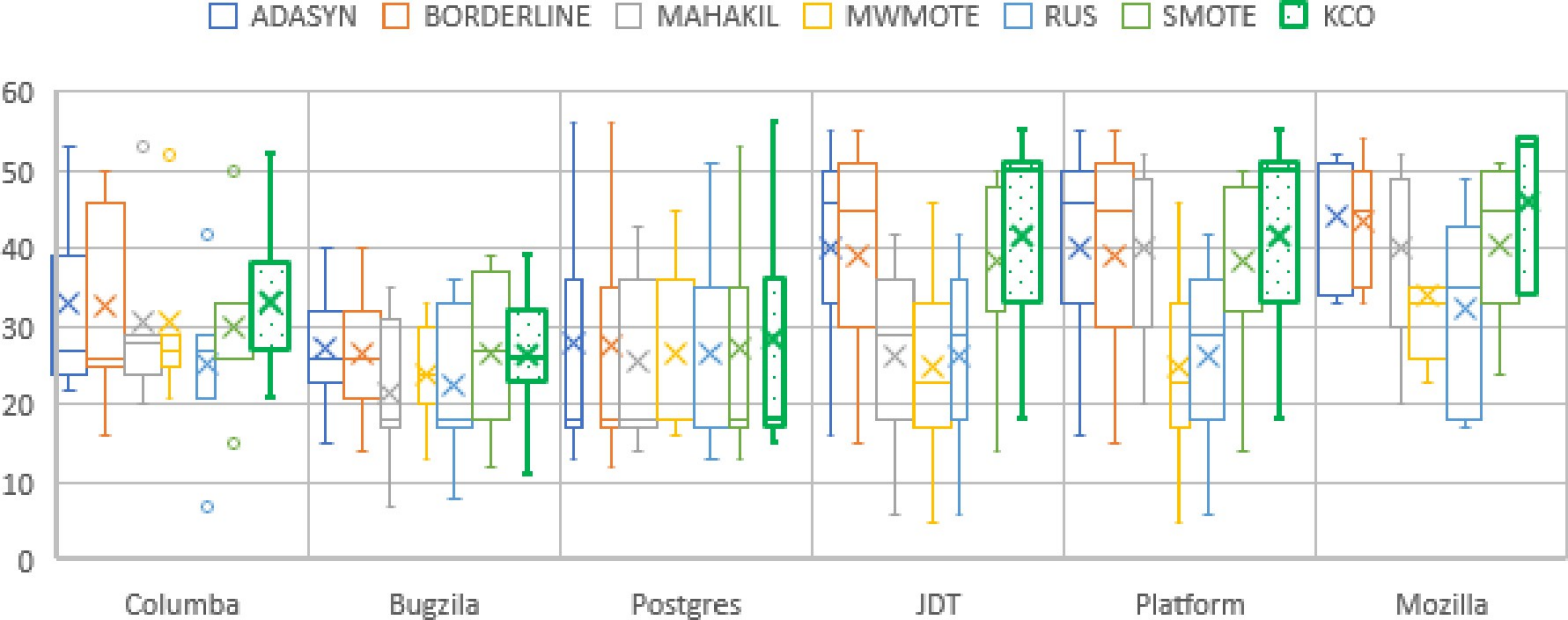
Resampling performance in cross project prediction.

The analysis further compares proposed KCO to the baseline techniques for cross project prediction as given in Table 4, and Fig 7. From the result, KCO achieves approximately in range of 33% to 46% across projects prediction for mean of F-score as given in Table 4. KCO outperforms or obtains similar performance to other baselines in almost all datasets, as achieves in the highest average score for JDT, Platform and Mozilla cross prediction. Contrary to other baseline techniques, none of the techniques achieves the highest average F-score. In exception for ADASYN and Borderline achieving draws in Columba, Bugzilla, and Postgres. Furthermore, MWMOTE, MAHAKIL and RUS are unable to produce substantially average in F1-score under cross project prediction setting. For cross project prediction, KCO demonstrates excellent performance in cross project prediction due to the consideration of the size of the data as an additional attribute for data resampling. Cross project prediction provides more information on the pattern of the defect class, resulting in a more diverse distribution. KCO takes advantage of the large size of defect instances and utilizes similarity analysis provided by KPCA to identify feasible regions for generating new samples. Resulting in a more accurate performance in cross project prediction, even when dealing with varying class distribution imbalance ratios in the original datasets. In essence, KCO leverages the benefits of a larger dataset size and the insights gained from the similarity analysis, which contribute to its superior performance in cross project predictions.

**Table 4:** Average of F-score for cross project prediction.

Techniques /Datasets	ADASYN	BORDERLINE	MAHAKIL	MWMOTE	RUS	SMOTE	KCO
Columba	**33**	**33**	31	31	25	30	**33**
Bugzilla	**27**	**27**	22	24	22	26	26
Postgres	**28**	**28**	26	26	27	27	**28**
JDT	40	39	26	25	26	38	**41**
Platform	40	39	40	25	26	38	**41**
Mozilla	44	43	40	34	33	41	**46**
Win/Draw/Lose	0/3/3	0/3/3	0/0/6	0/0/6	0/0/6	0/0/6	**3/2/1**

**Fig 8:**
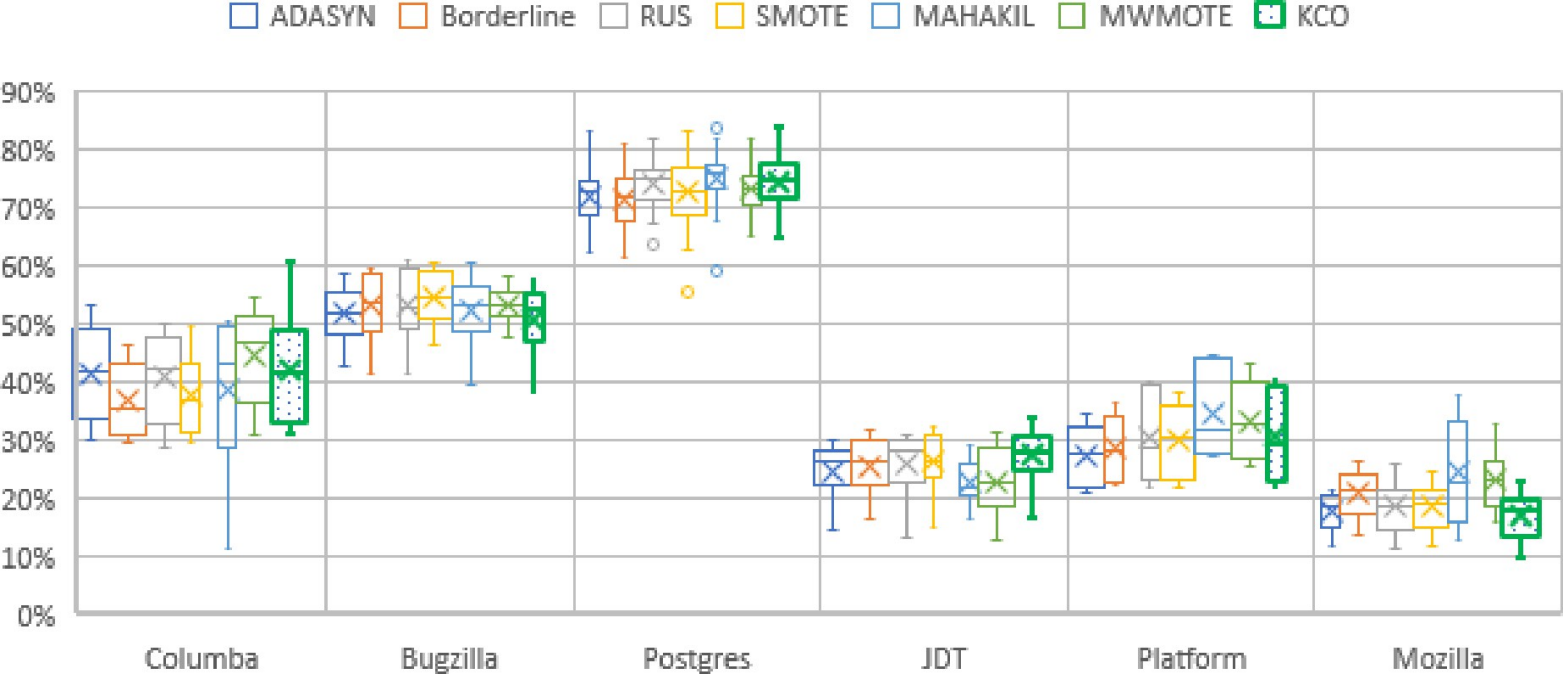
F-score of six datasets timewise predictions.

In Fig 8 and Table 5, we provide an evaluation of the prediction performance in the timewise validation scenario. The result indicates that the proposed technique KCO only obtained the highest average of F-scores for the JDT dataset. In contrast, MAHAKIL fared better in the Postgres, Platform, and Mozilla data sets, outperforming both KCO and other baseline techniques. KCO appears incapable of resampling effectively than other baseline techniques except for JDT datasets. The result differs from previous cross project prediction, where KCO performs significantly better than all the baseline methods when considering F-score. Despite the fact that KCO underperformed in timewise predictions, this only reflects the specificity rather than the generality of the technique performance. In terms of timewise prediction, KCO fails to achieve optimal results and performs worse than most baseline techniques. Since in KCO, the strength of data partitioning depends on the size of training data, and for each training fold consists of data of different sizes. Identifying suitable regions for interpolation faced difficulty in smaller data sets due to a lack of similarity among data samples. Accordingly, KCO appears to be insufficient as an appropriate technique for resampling smaller data sets. Note that proper hyperparameters for data partitioning in KCO will help to avoid this shortfall in smaller data sets.

**Table 5:** Average of F-score in timewise predictions.

Techniques/ Datasets	ADASYN	Borderline	RUS	SMOTE	MAHAKIL	MWMOTE	KCO
Columba	42	37	41	38	39	45	42
Bugzilla	52	53	53	55	52	53	51
Postgres	72	71	74	73	75	73	74
JDT	25	26	26	26	23	23	27
Platform	27	29	30	30	35	33	30
Mozilla	18	21	18	18	24	23	17

## 6. Threat of validity

A known validity of empirical experiments involves the quality of the data, which often difficult to obtain and verify. Nevertheless, noise and outliers inherent within datasets extracted from most open-source projects tend to have significant effects on prediction performance [40]. Removal of outliers from the original data presents a potential threat to these experimental results. The outliers introduce additional noise to the distribution of the original datasets. Nevertheless, oversampling techniques like MAHAKIL and MWMOTE possess the ability to handle outliers during data partition. Thus, the experiment conducted in this work exclude removal of outlier to produce a fair comparison. However, applications of data cleansing techniques for noise detection and elimination remain open for future investigations.

The software metrics considered for this analysis pose a potential threat to experimental results. By using a single set or type of metrics, generalization to other types of software metrics might not be valid for the reported results. Nonetheless, code and process metrics prove to perform very well and have proven useful in several empirical studies on JIT-SDP. The reason relates to the ease of extracting both types of metrics from any software once the VCS contains code change transactions.

The effectiveness of the proposed KCO depends on the ability to assess the diversity of data using KPCA. Despite KPCA’s benefits, high computation costs must be considered. In cases of large data sets with many features present, the covariance matrix proves difficult to calculate accurately. Thus, the initiative needs to allocate a significant amount of time and memory as these resources increase quadratically rather than linearly with the number of features. In the case of only a few features, however, the issue may not be significant. The challenge also applies to approaches based on Euclidean distances. The computation of covariance matrices for large dimensional features requires a more advanced and efficient method for handling the covariance aspect of training datasets.

## 7 Conclusions

This paper presents kernel based cross interpolation for oversampling the imbalanced class datasets in JIT-SDP. This study presented an experimental setup aimed at mitigating conclusion instability. We conducted an experiment to compare eight resampling techniques for developing JIT-SDP models derived from six state-of-the-art software projects. Although oversampling improves classification on average, a scatter distribution concentrated in local regions can be generated in extremely dense distribution datasets. Rather than being distributed evenly across the feature space, synthetic samples concentrate in local regions. Such a situation negatively impacts downstream resampling techniques that rely on accurate predictions of data cluster membership. Specifically, data partitioning techniques such as MAHAKIL and MWMOTE suffer from this problem. In light of this issue, KCO provides a diverse distribution of data by using a measure of similarity between data points to prevent the influence of nonlinear interaction between different attributes of samples. KCO uses kernel analysis to reduce the dimensionality of multivariate data while retaining maximum variation. Exploiting covariance among imbalanced data samples enables feasible interpolation spaces, reducing the impact of nonlinear distribution of imbalanced data. Additionally, KCO handles nonlinear data distributions in the datasets by crossover-interpolation to reduce near duplicate data for balanced class datasets. Crossover-interpolation produces generated data samples by multiple levels of pairing inheritance from the original data samples. As a result, KCO produces a more diverse set of data without compromising the origin information of the data distribution. Our work evaluates the performance of KCO on three different prediction settings. Experimental results show KCO consistently achieves higher F-score results for within-project and cross-project predictions. KCO achieves better overall classification performance, proving the feasibility of the approach in this study. Therefore, when dealing with an imbalanced class distribution task, KCO should be used for oversampling to improve JIT-SDP model classification performance. In future work, we plan to explore the impact of the different kernel functions in KPCA and the different activation functions in KCO on the performance of JIT-SDP models.

## Supporting information

S1 Datasets (ZIP)
